# Maternal and cord blood lipidomics as predictors of autism spectrum disorders: A systematic review

**DOI:** 10.1016/j.metop.2025.100403

**Published:** 2025-10-03

**Authors:** Antigoni Sarantaki, Ali Ghanchi, Joeri Vermeulen, Anastasia Barbouni, Ekaterina Charvalos, Aikaterini Sousamli, Dimitrios K. Anagnostopoulos

**Affiliations:** a Department of Midwifery, Faculty of Health & Care Sciences, University of West Attica, Egaleo, 12243, Greece; b Kauno Kolegija Higher Education Institution, Faculty of Medicine, Pramones pr 20, 50468, Kaunas, Lithuania; c Department of Life Sciences and Medicine, Faculty of Science, Technology and Medicine, University of Luxembourg, Esch-sur-Alzette, Grand Duchy of Luxembourg; d Department of Public Health, Biostatistics and Medical Informatics Research Group, Vrije Universiteit Brussel (VUB), Brussels, Belgium; e Department of Public and Community Health, School of Public Health, University of West Attica, Athens Campus, 11521, Greece; f European Health and Medical Consultants, EHMC, Girulių g. 20, Vilnius, LT-12123, Lithuania; g Section of Social Medicine -Psychiatry -Neurology, Athens Medical School, National & Kapodistrian University of Athens, 11527, Athens, Greece

**Keywords:** Autism spectrum disorder, Lipidomics, Pregnancy, Cord blood, Biomarker, Maternal, Predictors

## Abstract

**Background:**

Lipid metabolism is integral to neurodevelopment, contributing to neuronal membrane integrity, myelination, and signaling processes. Recent evidence indicates that disruptions in maternal and perinatal lipidomic profiles may be linked to an increased risk of autism spectrum disorders (ASD). To date, no systematic review has synthesized findings from human cohort studies examining lipidomic biomarkers during pregnancy or at birth in relation to subsequent ASD development.

**Methods:**

We systematically searched PubMed/MEDLINE, Embase, Scopus, Web of Science, Google Scholar, PsycINFO, CINAHL, and grey literature sources from inception to September 2025 for studies assessing maternal lipidomics during pregnancy, postpartum lipid profiles, or cord/neonatal lipidomics in relation to ASD diagnoses or autistic traits measured in offspring. Eligible study designs included prospective cohorts and nested case–control studies. Data extraction followed a standardized template, and methodological quality was appraised using the Newcastle–Ottawa Scale (NOS). Findings were synthesized narratively given heterogeneity in biospecimen timing, lipidomic platforms, and outcome measures. The protocol was registered with PROSPERO (CRD420251152074).

**Results:**

Nine prospective studies met the inclusion criteria. Maternal lipidomics during pregnancy indicated that lower ω-3 to ω-6 polyunsaturated fatty acid ratios and deficiencies in docosahexaenoic acid were associated with increased autistic traits or ASD with intellectual disability. Postpartum maternal lipid profiles showed that low low-density lipoprotein (LDL) cholesterol predicted greater ASD risk. Cord blood and neonatal lipidomics implicated acylcarnitines, sphingomyelins, and arachidonic acid–derived oxylipins in later ASD symptoms, with some studies demonstrating moderate predictive accuracy (AUROC ranging from 0.71 to 0.85) using machine learning approaches. Overall, recurrent disturbances in fatty acid metabolism, mitochondrial β-oxidation, and inflammatory lipid mediators were observed.

**Conclusions:**

Prospective evidence supports an association between maternal and neonatal lipidomic alterations and ASD risk, suggesting potential early biomarkers. However, heterogeneity across studies and reliance on single-timepoint measures limit comparability. Standardized lipidomic protocols, longitudinal sampling, and replication in diverse cohorts are needed to establish clinical utility and inform prevention strategies.

## 1 Introduction

**Fig. 1:**
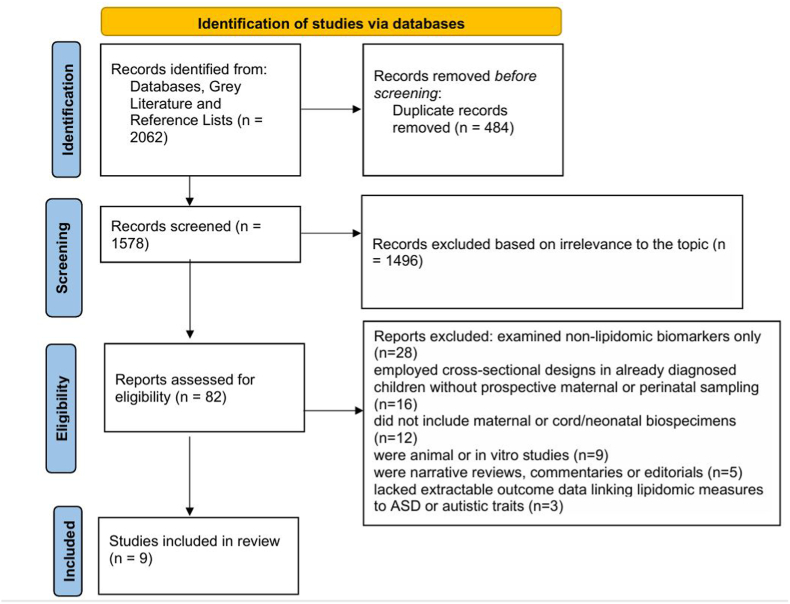
Illustrates the study selection process.

Lipidomics, the in-depth study of lipid species within biological systems, has emerged as a powerful tool in advancing precision medicine. Advances in high-resolution mass spectrometry and bioinformatics now allow the simultaneous quantification of hundreds to thousands of lipids across diverse classes, including phospholipids, sphingolipids, glycerolipids, sterols, and acylcarnitines [1]. As essential components of cell membranes, lipid mediators of signal transduction, and major energy substrates, lipids exert a wide range of effects on metabolism, immunity, and neurobiology [2]. Perturbations in lipid networks have been implicated in cardiovascular disease [3], respiratory infections [4], cancer [5], metabolic disorders [6], and increasingly, neurological and psychiatric conditions [7] (Fig. 1: PRISMA Flow Diagram of the Study Selection Process.)

During pregnancy, maternal lipid metabolism undergoes profound adaptations to support fetal growth and neurodevelopment. Polyunsaturated fatty acids (PUFAs) such as docosahexaenoic acid (DHA) and arachidonic acid (AA) are critical for neuronal membrane synthesis, synaptogenesis, and neurotransmission [8]. Altered maternal or placental lipid handling may therefore influence fetal brain development and long-term neurodevelopmental outcomes. Indeed, disorders of lipid metabolism, including cholesterol pathway abnormalities, have previously been linked to autism spectrum disorder (ASD) [9].

ASD is a heterogeneous neurodevelopmental condition characterized by impairments in social communication and restricted, repetitive behaviors, with an estimated prevalence of 1–2 % worldwide [10]. While early behavioral markers are well recognized, biological predictors measurable during pregnancy or the perinatal period remain poorly defined. Lipidomic profiling of maternal and cord blood provides an opportunity to identify molecular signatures that precede symptom onset, opening avenues for early risk stratification.

Emerging studies support this possibility. In a California population-based case–control study, lower mid-pregnancy ω-3 PUFA levels were associated with increased ASD risk, particularly in children with co-occurring intellectual disability [11]. Similarly, the Generation R cohort reported that a reduced maternal ω-3:ω-6 ratio in pregnancy correlated with higher autistic trait scores in childhood [12]. At the maternal–fetal interface, cord blood lipidomics have revealed distinct profiles associated with later ASD phenotypes, including acylcarnitine modules linked to early ASD symptoms [13] and AA–derived dihydroxy fatty acids correlated with greater ASD symptom severity and poorer adaptive function [14]. Furthermore, population-based analyses of neonatal dried blood spots have identified perturbations in acylcarnitine pathways in children later diagnosed with ASD [15,16].

Taken together, these findings suggest that maternal and perinatal lipidomic signatures may capture early biological pathways of ASD risk. This systematic review aims to synthesize current evidence on maternal and embryonic lipidomics in relation to ASD, evaluating their potential as early biomarkers and clarifying the consistency of lipid classes implicated in risk.

## 2 Materials and methods

This systematic review was conducted in accordance with the Preferred Reporting Items for Systematic Reviews and Meta-Analyses (PRISMA) 2020 guidelines [17]. The review protocol was registered in the International Prospective Register of Systematic Reviews (PROSPERO), with registration number CRD420251152074.

### 2.1 Search strategy

A comprehensive search strategy was developed in accordance with PRISMA recommendations to identify studies that investigated maternal or embryonic lipidomic profiles in relation to ASD. The electronic databases PubMed/MEDLINE, Embase, Scopus, Web of Science, Google Scholar, PsycINFO, and CINAHL were searched from inception to the most recent update in September 2025. The search strategy combined controlled vocabulary, such as MeSH and Emtree terms, with free-text keywords, encompassing three main concepts: autism, lipidomics, and maternal or embryonic biospecimens. An example of the PubMed query was: (“autism” OR “autism spectrum disorder” OR ASD OR autistic) AND (“lipidomics” OR “lipid profile” OR “fatty acid” OR PUFA OR triglyceride∗ OR phospholipid∗ OR sphingolipid∗ OR ceramide∗ OR acylcarnitine∗) AND (maternal OR pregnancy OR prenatal OR gestation OR “cord blood” OR neonatal OR perinatal OR embryo OR newborn). Equivalent search strings were adapted for each database to account for differences in indexing and syntax. No restrictions were applied with regard to publication year or language, and non-English articles were translated where feasible. To reduce publication bias, grey literature was also searched through ProQuest Dissertations and Theses, proceedings of relevant conferences including INSAR/IMFAR and the American Psychiatric Association, and clinical trial registries such as ClinicalTrials.gov and the WHO International Clinical Trials Registry Platform.

### 2.2 Inclusion and exclusion criteria

All studies identified through the search process were evaluated against predefined eligibility criteria based on the Population-Intervention-Comparison-Outcomes (PICO) framework. Eligible populations included mothers during pregnancy or immediately postpartum, as well as neonates and infants up to eighteen months of age, provided that lipidomic data were collected through maternal plasma, serum, or lipid fractions during gestation, or through cord blood collected at delivery, neonatal dried blood spots collected postnatally, or other perinatal biospecimens. The exposures of interest were lipidomic profiles measured using targeted or untargeted approaches, including analyses of PUFAs, phospholipids, sphingolipids, glycerolipids, ceramides, or acylcarnitines. ASD diagnosis was required to be established at ≥24 months of age, reflecting the minimum age for reliable diagnostic confirmation. Diagnoses at younger ages were excluded. No strict upper age limit was applied, and diagnoses made later in childhood (e.g., 3–6 years) were included if based on validated diagnostic criteria or standardized assessment tools.

To be eligible, studies were required to report subsequent autism spectrum disorder diagnosis, confirmed at a minimum of twenty-four months using DSM-IV or DSM-5 criteria, the Autism Diagnostic Observation Schedule, or multidisciplinary clinical assessment, or to report validated measures of autistic traits in early childhood as secondary outcomes.

We also included prospective cohort studies, nested case–control studies, and case–control studies embedded in longitudinal cohorts or biobank infrastructures. The exclusion criteria encompassed conference abstracts, narrative reviews, commentaries, and editorials. Furthermore, case series that lacked comparators, cross-sectional studies focusing solely on children with pre-existing diagnoses, and research involving animal models or in vitro methodologies were also omitted.

### 2.3 PRISMA process

A comprehensive search of databases from inception to September 2025 identified 1943 records. Grey-literature sources (ProQuest Dissertations and Theses, INSAR/IMFAR, APA conference proceedings, ClinicalTrials.gov, and WHO ICTRP) contributed 112 additional records. Manual screening of reference lists and relevant citations yielded a further seven records, resulting in 2062 records prior to deduplication. After removing duplicates, 1578 unique records remained for title/abstract screening. Of these, 1496 records were excluded as clearly irrelevant to the research question. Eighty-two articles were retrieved for full-text review. Seventy-three full texts were excluded for the following reasons: the study examined non-lipidomic biomarkers only (n = 28), employed cross-sectional designs in already diagnosed children without prospective maternal or perinatal sampling (n = 16), did not include maternal or cord/neonatal biospecimens (n = 12), were animal or in vitro studies (n = 9), were narrative reviews, commentaries or editorials (n = 5), or lacked extractable outcome data linking lipidomic measures to ASD or autistic traits (n = 3). Nine studies met all eligibility criteria and were included in the qualitative synthesis.

Title/abstract and full-text screening, as well as data extraction, were performed independently by two reviewers (A.S. and Aik.S.). Disagreements at any stage were first discussed between the reviewers, and unresolved discrepancies were adjudicated by a third reviewer (A.B.) to reach consensus. To ensure the robustness of the selection and extraction process, inter-reviewer agreement was quantified using Cohen's kappa (κ) statistic, which demonstrated substantial agreement for study inclusion decisions (κ = 0.82) and data extraction (κ = 0.79).

### 2.4 Risk of bias assessment

The methodological quality of included studies was evaluated independently by two reviewers using the Newcastle–Ottawa Scale (NOS) for cohort and case–control designs [18]. This tool assesses three domains: selection of participants, comparability of study groups, and ascertainment of outcomes. Each study could receive a maximum of nine stars, with higher scores reflecting lower risk of bias. Where appropriate, criteria were adapted for nested case–control studies and analyses conducted within population-based cohorts. Particular attention was given to the validity of ASD ascertainment, the timing and biological plausibility of lipidomic exposures, and the adequacy of adjustment for potential confounding factors. Disagreements in scoring were resolved through discussion and consensus, with a third reviewer consulted in cases of persistent discrepancy.

Across the nine included studies, NOS scores ranged from 6 to 9, indicating generally moderate to high methodological quality. Most studies scored highly on participant selection and outcome ascertainment but varied in comparability domains due to differences in adjustment for confounders such as maternal diet, metabolic status, and socioeconomic factors. Studies with lower NOS scores typically had smaller sample sizes, limited confounder adjustment, or relied on non-standardized outcome measures. In contrast, higher-scoring studies were generally large, population-based cohorts with comprehensive adjustment and validated diagnostic criteria, which increases confidence in their reported associations.

### 2.5 Data extraction

Data was extracted systematically into a predefined template. Information recorded included author and year of publication, country and cohort, study design, sample size, biospecimen type and timing, analytical platform, lipid classes or subclasses assessed, comparator groups, outcome definitions, and age at outcome assessment, main results with effect estimates, covariates used in statistical models, and the NOS quality score. Where studies reported both maternal and cord or neonatal lipidomics, results were extracted separately. In studies that examined both categorical ASD diagnoses and dimensional autistic traits, findings were summarized with specification of the outcome type.

A quantitative meta-analysis was not performed due to substantial methodological and clinical heterogeneity across the included studies. Differences existed in lipidomic platforms (ranging from targeted PUFA assays to untargeted high-resolution metabolomics), the number and type of lipid species profiled (from <20 to >800 species), biospecimen types (maternal serum vs. plasma, cord blood vs. dried blood spots), and sampling time points (mid-gestation, third trimester, delivery, neonatal period). Moreover, outcome measures varied markedly, including categorical ASD diagnoses, dimensional trait scales, and symptom severity indices. This variability precluded meaningful pooling of effect sizes and risk estimates without introducing significant bias. Future meta-analyses will become feasible once a larger body of studies adopts standardized lipidomic protocols, harmonized biospecimen collection windows, and comparable outcome definitions.

### 2.6 Data synthesis

Findings were synthesized qualitatively given the heterogeneity of study designs, biospecimen timing, lipidomic platforms, and outcome definitions. The synthesis was structured around the timing of biospecimen collection, distinguishing maternal lipidomics during pregnancy, postpartum maternal lipid profiles, cord blood lipidomics (collected at delivery), and early neonatal lipidomics (postnatal dried blood spots). Within each category, results were compared according to lipid classes, such as PUFAs, phospholipids, sphingolipids, ceramides, and acylcarnitines. Consistency of associations across studies was evaluated, and discrepancies were explored in light of methodological differences, analytic approaches, and outcome ascertainment. Where studies provided sufficient detail, subgroup findings, such as stratification by sex, co-occurring intellectual disability, or perinatal exposures, were also summarized. Given the limited number of studies and diversity of outcomes, a formal meta-analysis was not attempted. Instead, emphasis was placed on identifying recurrent lipidomic pathways implicated in ASD risk and highlighting gaps in the evidence base that warrant future research.

## 3 Results

### 3.1 Study characteristics

Nine eligible studies published between 2016 and 2024 were included [[11], [12], [13], [14],^16^,[19], [20], [21], [22]], encompassing large population-based cohorts, high-risk sibling cohorts, and biobank-linked case–control studies from Europe, North America, Asia, and Australia. Sample sizes ranged from fewer than 300 dyads in high-risk cohorts to over 9000 participants in population-based studies. Maternal biospecimens were collected either during pregnancy (mid-gestation or third trimester) or immediately postpartum, while perinatal samples consisted of cord blood collected at delivery or neonatal dried blood spots obtained in the first two weeks of life. Analytical platforms varied from targeted gas chromatography assays of PUFAs to high-resolution untargeted metabolomics capturing hundreds of lipid species, including phosphatidylcholines, sphingomyelins, ceramides, and acylcarnitines. Outcomes included clinically confirmed ASD diagnoses after two years of age or validated dimensional measures of autistic traits and symptoms assessed in early and middle childhood.

Table 1 summarizes the characteristics of the included studies.

**Table 1:** Summary of the characteristics of the included studies.

Author, Year	Country/Cohort	Study Design	Sample Size	Biospecimen & Timing	Analytical Platform	Lipid Classes	Comparator	Outcome & Age	Main Findings	Adjustments	NOS Score
Steenweg-de Graaff et al., 2016	Netherlands; Generation R (population-based birth cohort)	Prospective cohort	6999 maternal samples; 4624 mother–child pairs in outcome analysis	Maternal plasma glycerophospholipid fatty acids at ∼20.6 weeks' gestation	Gas chromatography of plasma glycerophospholipids	ω-3 PUFAs (ALA, EPA, DPA, DHA), ω-6 PUFAs (LA, AA, etc.), ω-3:ω-6 ratio	Higher vs. lower maternal PUFA levels/ratios	Autistic traits at 6 years, measured with SRS (short form) and CBCL pervasive developmental problems subscale	Lower ω-3:ω-6 ratio associated with more autistic traits (β = −0.008, 95 % CI: −0.016, −0.001). Higher total ω-6, especially linoleic acid, linked to more traits. No association for total ω-3 or individual ω-3. Maternal fish intake not associated.	Adjusted for maternal IQ, BMI, education, age, national origin, psychopathology, smoking, alcohol, folic acid, family income, paternal factors, breastfeeding, child age, sex, IQ	9/9
Huang et al., 2020	USA; MARBLES (Markers of Autism Risk in Babies – Learning Early Signs), high-risk siblings cohort	Prospective cohort	258 mother–child pairs (57 ASD, 62 non-typically developing, 139 TD)	Maternal plasma fatty acids (3rd trimester); dietary intake via FFQ (1st and 2nd half pregnancy)	Gas chromatography for plasma fatty acids; Block FFQ for dietary intake	ω-3 (ALA, EPA, DHA), ω-6 (LA, AA), total PUFA	ASD vs TD; non-typically developing vs TD	Clinical ASD diagnosis at 36 months (ADOS + DSM-IV criteria); non-typically developing classification based on MSEL and ADOS	Higher maternal total ω-3 intake in 2nd half pregnancy associated with 40 % reduced ASD risk (RR = 0.6, 95 % CI: 0.3–0.98). No associations for plasma ω-3/ω-6 in 3rd trimester with ASD. Higher plasma DHA and EPA associated with lower non-typically developing risk (RR = 0.93–0.99). Supplemental ω-3 intake not associated with ASD.	Models adjusted for maternal race, age, BMI, education, paternal age, gestational age, nutritional covariates (folate, vitamins, iron, calcium, etc.), storage/thaw variables	8/9
Langlois et al., 2020 [16]	USA; Texas Medicaid births (2007–2009) linked to Medicaid claims (2010–2012)	Matched case–control	3005 ASD cases, 6212 controls	Newborn dried blood spots (second screen, 1–2 weeks after birth)	Tandem mass spectrometry (MS/MS) of newborn screening analytes	Acylcarnitines (e.g., C8 octanoylcarnitine, C8/C2 octanoylcarnitine/acetylcarnitine, C6DC adipylcarnitine, C4DC methylmalonylcarnitine)	ASD vs non-ASD children	Clinical ASD diagnosis (ICD-9 codes 299.x) at 3–5 years via Medicaid claims	Higher octanoylcarnitine (C8) and C8/C2 associated with increased ASD risk (aOR 1.42–1.54 across total, term, and male births). Adipylcarnitine (C6DC) associated with higher ASD risk in second screen (aOR 1.29–1.33), but inverse asso		
Lyall et al., 2020 [11]	USA; California Prenatal Screening & Biobank (2010–2011 births)	Population-based case–control	499 ASD cases, 502 matched controls	Maternal serum at 15–19 weeks' gestation (2nd trimester)	Isotope dilution LC–HRMS (liquid chromatography–high-resolution mass spectrometry)	ω-3 (ALA, stearidonic acid, EPA, DPA, DHA), ω-6 (LA, GLA, DGLA, AA), total ω-3, total ω-6, total PUFA	ASD vs non-ASD children	ASD diagnosis from California DDS records at mean 5.2 years (range 4.1–6.2 years)	No strong overall associations between maternal PUFA levels and ASD. Modest inverse association with top quartile of linoleic acid (AOR = 0.74, 95 % CI: 0.49–1.11, P-trend = 0.10). Lowest decile of total and ω-3 PUFA associated with increased odds of ASD with comorbid intellectual disability (AOR = 2.78, 95 % CI: 1.13–6.82). No associations with ASD without ID. No evidence of effect modification by sex or ethnicity.	Adjusted for maternal age, race/ethnicity, education, prepregnancy BMI, health insurance, short interpregnancy interval	9/9
Park et al., 2021 [19]	USA; Boston Birth Cohort	Nested case–control within prospective cohort	88 ASD cases, 639 neurotypical controls	Maternal non-fasting plasma, collected 24–72 h postpartum	Clinical chemistry assays (CHOL, HDL, TG), LDL calculated (Friedewald equation)	Total cholesterol, HDL, LDL, triglycerides, LDL/HDL ratio	ASD vs neurotypical children	ASD defined via ICD-9 codes in EMR records (codes 299.xx), follow-up through 2015	One SD decrease in maternal LDL associated with higher ASD odds (aOR 1.35, 95 % CI: 1.04–1.75). Effect stronger in overweight/obese mothers (aOR 1.54, 95 % CI: 1.03–2.27). LDL <107 mg/dL (lowest tertile) linked to ∼2.5-fold higher ASD odds; in overweight/obese mothers, LDL <107 mg/dL associated with 4.6-fold higher odds (aOR 4.61, 95 % CI: 1.51–14.0). No consistent associations for HDL or TG.	Adjusted for gestational age, breastfeeding status, maternal race, sex, prepregnancy BMI, maternal age, birthweight, delivery type, pregestational and gestational diabetes	8/9
Che et al., 2023 [20]	Norway; Autism Birth Cohort (nested within MoBa)	Population-based nested case–control	408 MMG plasma (17–21 weeks gestation), 418 cord blood plasma	Maternal mid-gestation plasma; neonatal cord blood plasma	Untargeted metabolomics (GC–TOF MS, HILIC-QTOF MS, LC-QTOF MS), oxylipin panel	Complex lipids (phosphatidylcholines, sphingolipids, ceramides, triacylglycerols), fatty acids (AA, DHA, EPA), oxylipins	ASD vs non-ASD controls	ASD diagnosis (DSM-IV-TR, ADI-R, ADOS, ICD-10 NPR registry), follow-up ≥3 years	Dysregulation of multiple lipid classes. MMG: ASD girls showed reduced PC-PUFA and PE, but elevated galactosylceramides, ether-linked PCs. ASD boys: elevated homo-γ-linolenic acid, oxidized PC, lower DHA-derived 17-hydroxy-docosahexaenoic acid. CB: ASD boys had elevated arachidonic acid, higher AA/DHA ratio, reduced EpETrE and leukotriene B4. Machine learning AUROC 0.71–0.85 for prediction. Findings suggest inflammation, disrupted membrane integrity, altered neurotransmission.	Adjusted for maternal age, infections, autoimmune/allergic disorders, emotional distress (SCL-5), antipyretics, gestational age. Sensitivity analyses: folate, parental education, SSRI exposure.	9/9
Kaupper et al., 2023 [21]	Netherlands; Generation R Study	Prospective population-based cohort	783 cord blood samples; 716 with autistic trait data at 6 y, 648 at 13 y	Umbilical cord blood serum at birth	Targeted metabolomics (LC–MS/MS for amino acids, NEFAs, phospholipids, sphingomyelins, carnitines)	NEFAs, phospholipids (PCs, Lyso-PCs), sphingomyelins, carnitines	Associations within cohort, no clinical case–control	Autistic traits at ages 6 and 13 y measured with SRS (parent-reported)	Lower sphingomyelin SM.a.C39.2 and lower NEFA 16:1/16:0 ratio linked to higher autistic traits at 6 y in basic models (sex, age-adjusted) but attenuated after full adjustment (maternal BMI, smoking, education, psychopathology, alcohol, gestational age, birthweight). Carnitine C18:2 showed a trend toward higher autistic traits but not significant after FDR correction. No associations at 13 y.	Adjusted for maternal BMI, psychopathology, education, smoking, alcohol, gestational age, birthweight, folate and vitamin D sensitivity analyses	9/9
Vacy et al., 2024 [13]	Australia; Barwon Infant Study (BIS)	Prospective population-based birth cohort	920 cord blood samples; 587 with ASD/ADHD symptom data	Cord blood serum at birth (UHPLC–MS/MS lipidomics); maternal serum at 28 wks gestation and child blood at 6 mo, 12 mo, 4 y also profiled	UHPLC–MS/MS lipidomics, 776 lipid features across 36 classes, networked via WGCNA	Acylcarnitines, phosphatidylethanolamines, phosphatidylserines, triglycerides, sphingomyelins, lysophosphatidylcholines, cholesteryl esters	Internal cohort comparisons (continuous lipidome vs symptom scores)	ASD and ADHD symptoms at 2 y (Child Behavior Checklist DSM-5 autism and ADHD subscales); replication at 4 y with SDQ	Elevated acylcarnitine module (Cyan-AC) strongly predicted increased ASD and ADHD symptoms at age 2 (β = 0.38, 95 % CI 0.19–0.57 for ASD; β = 0.54, 95 % CI 0.33–0.75 for ADHD per 1 SD increase). Associations persisted after FDR correction and extensive confounder adjustment. Cyan-AC mediated 12 % of the effect of low household income on ASD symptoms and 16 % of the effect of low Apgar score on ADHD symptoms. Other lipid modules (PE, PS, TG) also associated but effects attenuated when Cyan-AC included. Findings replicated for ADHD/ASD-related traits at 4 y.	Adjusted for child sex, gestational age, maternal contamination, storage variables, maternal age, income, smoking, stress, and metabolic markers (NOPMS, GlycA). IPW used for selection bias.	9/9
Hirai et al., 2024 [14]	Japan; Hamamatsu Birth Cohort (HBC)	Prospective cohort (ASD oversampled subset)	200 children (cord blood + ASD symptom assessment)	Umbilical cord blood serum, collected at birth	Targeted LC–MS/MS for CYP-PUFA metabolites (EpFAs, diHETrEs, etc.)	Arachidonic acid-derived dihydroxy fatty acids (diHETrEs: 8,9-; 11,12-; 14,15-), epoxy fatty acids (EETs)	Internal comparisons within cohort	ASD symptoms (ADOS-2 Calibrated Severity Score) and adaptive functioning (VABS-II) at age 6 years	Higher 11,12-diHETrE, 14,15-diHETrE, and total diHETrE were significantly associated with more severe ASD symptoms (ADOS-2 CSS β = 0.251, P = 0.0003). Sex-specific: in girls, higher 8,9- and 11,12-diHETrE linked to ASD severity; in boys, 14,15-diHETrE. Elevated 11,12-diHETrE associated with poorer socialization/coping skills on VABS-II. Findings implicate proinflammatory diHETrE in ASD pathophysiology.	Adjusted for maternal BMI (late pregnancy), maternal age, gestational age, birthweight, sex, parity, delivery method	9/9

AA = Arachidonic acid; ADHD = Attention-deficit/hyperactivity disorder; ADI-R = Autism Diagnostic Interview–Revised; ADOS = Autism Diagnostic Observation Schedule; ADOS-2 CSS = Autism Diagnostic Observation Schedule, Second Edition – Calibrated Severity Score; ASD = Autism spectrum disorder; aOR = Adjusted odds ratio; ALA = Alpha-linolenic acid; AUROC = Area under the receiver operating characteristic curve; BIS = Barwon Infant Study; BMI = Body mass index; CB = Cord blood; CBCL = Child Behavior Checklist; CHOL = Total cholesterol; CI = Confidence interval; C6DC = Adipylcarnitine; C8 = Octanoylcarnitine; C8/C2 = Octanoylcarnitine/acetylcarnitine ratio; C4DC = Methylmalonylcarnitine; DDS = Department of Developmental Services (California); DHA = Docosahexaenoic acid; diHETrE = Dihydroxyeicosatrienoic acid; DGLA = Dihomo-gamma-linolenic acid; DPA = Docosapentaenoic acid; DSM-IV = Diagnostic and Statistical Manual of Mental Disorders, Fourth Edition; DSM-IV-TR = Diagnostic and Statistical Manual of Mental Disorders, Fourth Edition, Text Revision; DSM-5 = Diagnostic and Statistical Manual of Mental Disorders, Fifth Edition; EETs = Epoxyeicosatrienoic acids; EMR = Electronic medical record; EPA = Eicosapentaenoic acid; EpETrE = Epoxyeicosatetraenoic acid; EpFAs = Epoxy fatty acids; FDR = False discovery rate; FFQ = Food frequency questionnaire; GC = Gas chromatography; GC–TOF MS = Gas chromatography–time of flight mass spectrometry; GLA = Gamma-linolenic acid; GlycA = Glycoprotein acetyls (systemic inflammation marker); HBC = Hamamatsu Birth Cohort; HDL = High-density lipoprotein cholesterol; HILIC-QTOF MS = Hydrophilic interaction liquid chromatography–quadrupole time-of-flight mass spectrometry; HRMS = High-resolution mass spectrometry; ICD-9 = International Classification of Diseases, Ninth Revision; ID = Intellectual disability; IPW = Inverse probability weighting; IQ = Intelligence quotient; LA = Linoleic acid; LC–HRMS = Liquid chromatography–high-resolution mass spectrometry; LC–MS/MS = Liquid chromatography–tandem mass spectrometry; LDL = Low-density lipoprotein cholesterol; Lyso-PC = Lysophosphatidylcholine; MARBLES = Markers of Autism Risk in Babies – Learning Early Signs; MoBa = Norwegian Mother, Father and Child Cohort Study; MMG = Maternal mid-gestation; MS/MS = Tandem mass spectrometry; MSEL = Mullen Scales of Early Learning; NEFA = Non-esterified fatty acids; NOPMS = N-acylphosphatidylethanolamines (lipid marker, Barwon study); NOS = Newcastle–Ottawa Scale; PC = Phosphatidylcholine; PC-PUFA = Phosphatidylcholine–polyunsaturated fatty acids; PE = Phosphatidylethanolamine; PS = Phosphatidylserine; PUFA = Polyunsaturated fatty acid; RR = Relative risk; SCL-5 = Hopkins Symptom Checklist-5 (maternal distress scale); SD = Standard deviation; SDQ = Strengths and Difficulties Questionnaire; SM = Sphingomyelin; SRS = Social Responsiveness Scale; SSRI = Selective serotonin reuptake inhibitor; TG = Triglycerides; TD = Typically developing; VABS-II = Vineland Adaptive Behavior Scales, Second Edition; WGCNA = Weighted gene co-expression network analysis; ω-3 = Omega-3 fatty acids; ω-6 = Omega-6 fatty acids.

### 3.2 Maternal lipidomics and ASD risk

Four studies analyzed maternal lipidomic profiles during pregnancy. Two targeted PUFA assays showed converging evidence for an unfavorable balance of maternal fatty acids in relation to autistic outcomes. Within the Generation R cohort, a lower ω-3:ω-6 ratio during mid-pregnancy, coupled with elevated levels of linoleic acid, was correlated with an increase in autistic traits observed at the age of six [12]. In the MARBLES high-risk cohort, higher dietary ω-3 intake during late pregnancy was linked to a 40 % reduction in ASD risk, although maternal plasma PUFA concentrations in the third trimester were not associated with clinical ASD [22]. A large population-based case–control study in California reported that extremely low levels of maternal ω-3 and total PUFA in mid-pregnancy serum increased the odds of ASD with comorbid intellectual disability nearly three-fold, though no associations were observed for ASD without intellectual disability [11]. In the most comprehensive metabolomic study to date, conducted as part of the Norwegian Autism Birth Cohort, researchers identified significant changes in lipid classes, such as phosphatidylcholines, ether-linked phospholipids, and ceramides, with distinct patterns based on sex. By integrating these maternal biomarkers, predictive models achieved an area under the receiver operating characteristic curve (AUROC) of up to 0.71 for predicting future ASD [20]. Collectively, these findings suggest that imbalances in maternal fatty acid composition and dysregulated lipid metabolism during gestation may confer increased risk for ASD, though results vary by lipid class, timing of measurement, and case definition.

### 3.3 Postpartum maternal lipid profiles

One study investigated postpartum maternal lipid status. In the Boston Birth Cohort, low maternal LDL cholesterol measured within 72 h of delivery was associated with higher odds of ASD in the offspring, with the effect amplified among overweight and obese mothers [19]. These findings indicate that maternal lipid dysregulation persisting into the immediate postnatal period may reflect metabolic states relevant to offspring neurodevelopment.

### 3.4 Cord blood and early neonatal lipidomics

Five studies assessed cord blood collected at birth or early neonatal lipidomics from dried blood spots collected postnatally. In the Barwon Infant Study, network analysis of the cord blood lipidome revealed that an acylcarnitine-enriched module strongly predicted ASD and ADHD symptoms at age two, with replication at age four, and mediated part of the association between adverse perinatal exposures and neurodevelopmental traits [13]. Similarly, the Generation R cohort found that lower sphingomyelin concentrations and altered non-esterified fatty acid ratios in cord blood were associated with higher autistic traits at age six, although these associations attenuated after adjustment for confounders [21]. A Japanese birth cohort reported that elevated AA–derived dihydroxy fatty acids in cord blood were linked to more severe ASD symptoms and poorer adaptive functioning at age six, with sex-specific patterns [14]. Complementing these findings, a large case–control study in Texas demonstrated that altered neonatal acylcarnitines measured from newborn screening dried blood spots were associated with later ASD diagnoses based on Medicaid claims, with increased risk observed for octanoylcarnitine and related species [16]. Finally, in the Norwegian Autism Birth Cohort, cord blood lipidomic profiles distinguished ASD cases from controls with AUROC values up to 0.85, with sex-differentiated signals including elevated AA and altered oxylipin balance in male cases [20].

## 4 Discussion

This systematic review identified nine prospective studies investigating maternal and perinatal lipidomic profiles in relation to ASD. Despite heterogeneity in study designs and analytic platforms, several consistent findings emerged. Maternal lipid imbalances during pregnancy, particularly low ω-3:ω-6 ratios and deficiencies in polyunsaturated fatty acids (PUFAs), were associated with increased risk of autistic traits or ASD, with some evidence that extremely low maternal ω-3 levels specifically predicted ASD with comorbid intellectual disability [11,12]. At birth and early postnatal periods, cord blood and neonatal lipidomics identified perturbations in acylcarnitines, sphingomyelins, and oxylipins as correlates of later ASD symptoms or diagnoses [13,14,16]. Untargeted lipidomics further revealed broad alterations in phosphatidylcholines, ceramides, and fatty acid–derived mediators with predictive value for ASD in population-based cohorts [20]. Taken together, these findings suggest that disturbances in maternal and perinatal lipid metabolism may represent early biological signatures of ASD risk.

To explore the robustness of our conclusions, we qualitatively examined whether key lipidomic signals persisted among the highest-quality studies (NOS ≥8). This sensitivity narrative revealed that the most consistent associations—such as lower maternal ω-3:ω-6 PUFA ratios during pregnancy and elevated neonatal acylcarnitine levels—were present in nearly all high-scoring studies [[11], [12], [13],^20^]. Similarly, the strongest metabolomic predictive models based on phosphatidylcholines, ceramides, and oxylipins were derived from well-designed prospective cohorts with rigorous outcome ascertainment [20]. Conversely, more heterogeneous or inconsistent findings, such as those related to postpartum maternal lipid profiles [19] or specific oxylipin subtypes [14], were often reported by studies with lower methodological quality. This pattern supports the conclusion that the core lipidomic alterations identified in this review are unlikely to be artefacts of study bias and may reflect biologically meaningful pathways underlying ASD risk.

A key source of heterogeneity across studies relates to the timing of biospecimen collection and the analytical strategies employed. Maternal lipidomic profiles reflect systemic metabolic and dietary influences during gestation and may capture upstream risk signals that precede neurodevelopmental alterations. In contrast, cord blood and neonatal lipidomics represent the fetal or newborn metabolic milieu at the maternal–fetal interface and thus may be more proximal indicators of neurodevelopmental outcomes. Indeed, cord blood acylcarnitines and oxylipins showed stronger predictive performance (AUROC values up to 0.85) compared with maternal PUFA ratios (AUROC ≤0.71) [13,20], suggesting that perinatal lipid profiles might offer superior discriminative capacity for early ASD risk. However, these findings should be interpreted in the context of methodological diversity: targeted assays typically focused on a limited set of known biomarkers, whereas untargeted platforms provided broader but more heterogeneous signatures. This variability underscores the importance of harmonizing both sampling windows and analytical techniques in future research to enable direct comparisons of biomarker performance.

Lipids are fundamental to brain development, serving as structural components of neuronal membranes, substrates for myelination, and precursors of signaling molecules [8,23]. Deficits in ω-3 fatty acids such as docosahexaenoic acid (DHA) impair synaptic plasticity and neurotransmission, while excess ω-6 fatty acids may promote pro-inflammatory eicosanoid production [24]. The consistent associations of acylcarnitine dysregulation with ASD across multiple studies highlight a potential role of mitochondrial dysfunction and impaired β-oxidation, which have been reported in subsets of children with ASD [25]. Moreover, cord blood oxylipin profiles implicating elevated AA–derived dihydroxy fatty acids suggest activation of inflammatory lipid mediator pathways during the perinatal period [14]. Collectively, these mechanistic pathways converge on neuroinflammation, disrupted energy metabolism, and impaired neuronal connectivity, providing biologically plausible links between lipid dysregulation and atypical neurodevelopment.

It is crucial to distinguish between lipidomic alterations as *biomarkers* of ASD risk and their potential *etiologic* roles. Most included studies were observational, and associations do not establish causality. Lipidomic signatures—such as altered acylcarnitine patterns or PUFA imbalances—may reflect downstream consequences of other risk factors (e.g., maternal inflammation, placental function, or genetic susceptibility) rather than direct causal mechanisms. As such, these findings are best interpreted as markers of biological processes linked to atypical neurodevelopment, rather than definitive drivers of ASD pathogenesis. Experimental and mechanistic studies, including Mendelian randomization and interventional trials, will be necessary to clarify whether modulating these lipid pathways can alter neurodevelopmental outcomes.

The findings of this review underscore the potential of maternal, cord blood, and early neonatal lipidomic profiling as a component of early risk stratification for ASD. Targeted PUFA assays could inform dietary or supplementation interventions during pregnancy, as suggested by observational evidence linking higher maternal ω-3 intake with reduced ASD risk [22]. More comprehensive lipidomic signatures, particularly when combined with machine learning approaches, demonstrated moderate predictive ability [20], raising the possibility of developing biomarker-based screening tools. Integration of lipidomics with genetic, epigenetic, and microbiome data may further enhance predictive accuracy. However, clinical translation requires replication across diverse populations, establishment of robust thresholds, and demonstration that lipid-targeted interventions can alter neurodevelopmental trajectories.

This review is the first to comprehensively evaluate evidence from human studies linking maternal or neonatal lipidomics to later ASD outcomes. A major strength of the included studies is their prospective design, with lipidomic data collected during pregnancy or at birth, before ASD onset. Another strength of this review was the comprehensiveness of the search strategy, which encompassed multiple electronic databases as well as grey literature sources, thereby reducing the risk of missing relevant studies. Several cohorts were large and population-based, reducing selection bias and allowing detailed adjustment for confounders. The application of both targeted and untargeted lipidomics expanded understanding from individual fatty acids to complex lipid networks.

Nonetheless, important limitations must be acknowledged. Outcome heterogeneity was notable, with some studies relying on clinical diagnoses and others on dimensional trait measures. Biospecimen timing varied, and most studies measured lipids at a single time point, limiting inference about dynamic changes across gestation and infancy. Differences in analytical platforms and statistical handling of high-dimensional lipidomic data complicate comparability. Finally, potential residual confounding by diet, genetics, or co-exposures such as maternal metabolic disorders cannot be excluded. Another limitation is the possibility of publication bias. Although our comprehensive search strategy included grey literature and conference proceedings, the small number and heterogeneity of studies prevented a formal statistical assessment (e.g., funnel plot).

Another important limitation is the potential for publication bias, as studies reporting null or negative findings may be underrepresented despite our efforts to include grey literature and conference proceedings. This bias could lead to overestimation of associations between lipidomic signatures and ASD risk. Furthermore, integrating lipidomic data with other omics layers—such as genomics, transcriptomics, proteomics, and microbiome profiles—poses substantial analytical and methodological challenges. Differences in data dimensionality, normalization, and statistical frameworks complicate multi-omic integration and limit the ability to delineate causal pathways. Future research should prioritize standardized analytical pipelines and cross-platform harmonization to enable robust multi-omic analyses and improve mechanistic interpretation.

It is important to note that the associations identified in this review cannot be interpreted as causal. Observational designs are inherently subject to residual confounding, effect modification, and complex interactions among genetic, dietary, metabolic, and environmental factors. These influences may partially explain the observed lipidomic signatures and underscore the need for caution in attributing causality. Advanced causal inference methods and mechanistic studies will be essential to disentangle whether lipid alterations represent biomarkers of risk or play a direct etiological role in ASD development.

While these findings raise the possibility of lipid-targeted prevention strategies—such as maternal ω-3 supplementation or dietary modulation—several feasibility and evidence gaps remain. Optimal timing of intervention is not yet defined; it is unclear whether modifying maternal lipid status preconceptionally, during early gestation, or later in pregnancy is most effective for influencing fetal neurodevelopment. Moreover, existing evidence for ω-3 supplementation in reducing ASD risk is inconsistent and largely derived from observational studies, with no large-scale randomized trials demonstrating efficacy. Practical considerations, including individual variability in lipid metabolism, adherence, and safety in specific populations, also warrant careful evaluation. Before lipidomic biomarkers can inform clinical decision-making or screening algorithms, they require validation in diverse populations, establishment of clinically actionable thresholds, and demonstration that modifying lipid profiles leads to measurable neurodevelopmental benefits.

Future reviews, once a larger and more homogeneous evidence base is available, should address this issue. These limitations highlight the need for standardized lipidomic methodologies, longitudinal sampling, and harmonized outcome assessments in future research. In forthcoming research on lipidomics and ASD, it is imperative to prioritize the harmonization of methodologies and foster interdisciplinary collaboration. Establishing standardized protocols for the collection, storage, and analysis of biospecimens is essential to improve data comparability across diverse cohorts.

Longitudinal sampling during pregnancy and infancy is crucial to capture the dynamic changes in lipid metabolism and their temporal association with neurodevelopment. Furthermore, lipidomic studies should increasingly be incorporated into large, ethnically diverse population-based cohorts to improve generalizability and enable stratified analyses based on sex, maternal metabolic status, and co-occurring conditions. Additionally, the integration of lipidomic profiles with genomic, epigenomic, and microbiome data could elucidate multi-omic pathways underlying ASD risk. Causal inference methodologies, such as Mendelian randomization and mediation analyses, may provide clarity on whether lipid alterations serve as biomarkers of risk or mechanistic contributors to ASD development. Ultimately, clinical translation will rely on demonstrating the feasibility of incorporating lipidomic biomarkers into early screening frameworks and targeting modifiable lipid profiles, particularly PUFA status, through nutritional or metabolic interventions to mitigate the risk of ASD.

## 5 Conclusions

This systematic review demonstrates that disturbances in maternal, cord blood, and early neonatal lipid metabolism, including imbalances in PUFAs, alterations in phospholipids and sphingolipids, and dysregulated acylcarnitine and oxylipin pathways, are consistently associated with ASD risk or related phenotypes. While findings across diverse cohorts provide biologically plausible evidence linking lipidomic profiles to early neurodevelopmental trajectories, heterogeneity in study designs, biospecimen timing, and analytic methods limits direct comparability. Lipidomics holds promise as a tool for identifying early biomarkers of ASD. Still, validation in larger, harmonized, and ethnically diverse cohorts, alongside mechanistic and interventional studies, is essential before clinical translation into predictive screening or preventive strategies can be realized.

## CRediT authorship contribution statement

**Antigoni Sarantaki:** Writing – review & editing, Writing – original draft, Visualization, Methodology, Investigation, Formal analysis, Data curation, Conceptualization. **Ali Ghanchi:** Writing – review & editing, Validation. **Joeri Vermeulen:** Writing – review & editing, Formal analysis. **Anastasia Barbouni:** Writing – review & editing, Methodology. **Ekaterina Charvalos:** Visualization, Validation, Investigation. **Aikaterini Sousamli:** Software, Investigation, Data curation. **Dimitrios K. Anagnostopoulos:** Validation, Supervision.

## Conflict-of-interest/financial disclosure statement

The authors declare that they have no known competing financial interests or personal relationships that could have appeared to influence the work reported in this paper. No specific funding was received for this study.
